# Role of Genital Tract Bacteria in Promoting Endometrial Health in Cattle

**DOI:** 10.3390/microorganisms10112238

**Published:** 2022-11-12

**Authors:** Mounir Adnane, Aspinas Chapwanya

**Affiliations:** 1 Department of Biomedicine, Institute of Veterinary Sciences, University of Tiaret, Tiaret 14000, Algeria; 2 Department of Clinical Sciences, Ross University School of Veterinary Medicine, Basseterre 00265, Saint Kitts and Nevis

**Keywords:** inflammation, endometrial microbiome, cyclicity, probiotic, breeding, postpartum uterine disease

## Abstract

Microbiota regulate endometrial health in cattle. It is important to know what a ‘good’ microbiome is, in order to understand pathogeneses of uterine disease. Given that microbial influx into the genital tract of cows at calving is unavoidable, exploring the involvement of genital tract bacteria in promoting endometrial health is warranted. The dysbiosis of endometrial microbiota is associated with benign and malign uterine diseases. The present review discusses current knowledge about the altered endometrial microbiome and the implications of this modulation on endometrial inflammation, ovarian activity, fecundation, pregnancy, and postpartum complications. Intravaginal administration of symbiotic microbes in cattle is a realistic alternative to antibiotic and hormone therapy to treat uterine disease. Genital microbial diversity can be modeled by nutrition, as the energy balance would improve the growth of specific microbial populations. It may be that probiotics that alter the endometrial microbiome could provide viable alternatives to existing therapies for uterine disease in cattle.

## 1. Introduction

Mammalian organs and systems harbor a complex yet dynamic microbiome, especially genital and digestive tracts. In cattle, the genital tract microbiome exerts a profound influence on endometrial health, homeostasis, and fertility. Many factors contribute to the colonization of the uterus by a diverse microbiome. The gut is considered as one of the main drivers of bacteria, viruses, and protozoa that colonize the genital tract [1]. Gut microbiota provide essential volatile fatty acids and amino acids that were implicated in sexual communication in mice [2,3]. Rumen microbiota produce enzymes to break down complex feed molecules into simpler ones assimilable in the intestines. In turn, the host provides optimal condition for growth and proliferation of the microbes [4]. We believe that a similar relationship exists in the genital tract of cattle. Biomolecules such as vaginal mucus or host defense proteins play a crucial role in modulating immune responses to these microorganisms. The genital tract microbiota offer many benefits to the host, through a range of physiological functions such as maintaining endometrial epithelial integrity, protecting against pathogens, and regulating host immunity [5,6,7]. For instance, in women, *Lactobacillus* abundant in the vagina protect the vaginal mucosa by preventing pathogen adhesion through a competition effect [7,8]. In this symbiotic relationship, *Lactobacillus* utilize genital tract secretions such as mucin carbohydrates as a source of nutrition. In turn, *Lactobacillus* secrete immune active molecules such as lactic acid and hydrogen peroxide that prevent pathogenic bacterial proliferation [9,10]. Recent advances in microbiology now allow identification of all bacteria present in tissues or organs to the level of phyla or genus. Gene analysis based on the well-conserved 16S ribosomal ribonucleic acid (rRNA) region allowed to accurately detect microbes in organs such as the pregnant uterus previously thought sterile. Bacteria were detected in the uterus of virgin heifers and pregnant cows that were clinically healthy with no risk or gestational complications [11]. Vaginal and uterine microbiome load and diversity are highly dynamic and are influenced by factors such as origin of contamination, delivery mode, nutrition, and postpartum complications [12]. Fecal matter contamination and vaginal microbiomes are the main sources for endometrial colonization contributing to the normal genital microbiome of cows and possible infections [1,5,13]. The sequencing of the V3–V4 region of the 16S rRNA of ectocervicovaginal lavage revealed that the normal vaginal microbiome comprises bacterial phyla *Bacteroidetes*, *Fusobacteria*, and *Proteobacteria*, in association with Archaeal order *Desulfurococcales* [14,15]. *Lactobacillus* spp. are present at low rates in 90% of cows postpartum [14,16]. The natural microbiome is beneficial to the host by producing a biofilm, in association with the host secretions such as vaginal mucus that protect the underlying tissue from pathogen invasion [14]. *Lactobacillus* can co-aggregate with pathogens, which prevent them from adhering to their receptors or ligands in the host’s mucosa. In addition, some of these bacteria produce lactic acid, which reduces vaginal pH and thereby interferes with pathogen proliferation and survival [17]. At normal concentration, lactic acid can inactivate, in vitro, different pathogens, including *Chlamydia trachomatis*, *Neisseria gonorrhoeae*, and *Escherichia coli (E.coli)* [18,19,20]. Changes of the natural balance within populations of the genital microbiome led to dysbiosis and genital diseases which compromise female’s fertility [15,21,22,23,24]. For instance, *Gardnerella vaginalis,* highly pathogenic bacteria, produce extracellular sialidase, a powerful enzyme that hydrolyzes sialic acid from sialoglycoproteins in bioactive molecules such as secreted Immunoglobulin A (IgA), therefore interfering with the efficiency of the immune system and increasing the risk of infection with other pathogens [25]. The presence of *Chlamydia trachomatis*, *Gardnerella vaginalis*, *Ureaplasma species*, and Gram-negative stains in women’s genital tract would disrupt fertility [24]. Studies report that many factors affect microbial load and diversity; however, few are those describing how the microbiome affects animal fertility. It is known that pheromones which play a vital role in mating are produced, in part, by the vaginal microbiome of different species, including cows [26,27]. In the present paper we review the interaction of genital microbiomes with the host tissues. We aim to explore how cattle fertility is affected by the genital microbiome and suggest possible strategies to improve reproduction and health management, including the use of prebiotics and probiotics. The present paper is not a systematic review; however, search and selection of implicated references were based on relevance to the topic and prioritizing recent published papers.

## 2. Genital Microbiome and Modulation of Uterine Inflammation

During calving, dilatation of the cervix results in bacterial influx and contamination of the uterus. For cows that develop endometritis, the bacteria present in the uterus postpartum may either be the etiological agents or secondary infections [28]. Molecular-based methods targeting the 16S rRNA gene in vaginal and uterine samples revealed that the high prevalence of pathogenic bacteria such as *Fusobacterium* and *Corynebacterium* was commonly associated with metritis, endometritis, and infertility [29,30]. However, the presence of other bacterial species is crucial in the regulation of endometrial inflammation. For instance, *Lactobacillus* interferes with the secretion of proinflammatory cytokines stimulated by *E. coli* [31]. In vivo, combining *Lactobacillus* and *Pediococcus* cultures resulted in robust control of an endometrial inflammatory response by *E. coli* [32]. In addition, *Lactobacillus sp* produce bioactive molecules such as lactic acid and hydrogen peroxide, which inhibit growth of *Staphylococcus aureus* (*S. aureus*) and *Trueperella pyogenes* (*T. pyogenes*) that are commonly isolated from cattle suffering from uterine disease [33,34]. Lactic acid is a powerful acidic substance that can reach into sensitive microbes without specific receptors and increase cytosol acidity, leading to bacterial death [35]. Thus, a strategy of using *Lactobacillus* as a probiotic peripartum would likely reduce postpartum uterine disease and improve fertility.

## 3. Genital Microbiome and Cyclicity

It is well documented that endocrine hormones controlling the estrous cyclicity of cows influence the genital tract microbiome as well as its diversity [36]. In turn, genital microbiota modulates reproductive cycle hormonal profiles [37]. During the follicular phase, high estradiol concentrations lower the pH of endometrial secretions [38]. Thus, estradiol alters microbial diversity in the vagina during the follicular phase [39]. Therefore, it seems that the microbiome present during a specific time influences the estrous cyclicity and quality of developing oocytes [2,37]. Similarly, when present *Lactobacillus* decreases the vaginal pH and promotes reproductive function by suppressing infectious pathogens, improving oocyte quality and promoting luteal function [2,40,41]. During disease, if the uterus is infected by lipopolysaccharide (LPS)-producing bacteria, such as *E. coli*, an important inflammatory reaction is triggered in the endometrial tissue and LPS is accumulated in the antrum [42,43]. Furthermore, granulosa cells have specific receptors for LPS, Toll-like receptors 4 (TLR-4), which recognize and respond to LPS, thus triggering an inflammatory reaction in the follicular cells which compromise steroidogenic activity and the development of oocytes through the inhibition of mitotic activity [44,45]. Clinical studies have confirmed that severe uterine contamination was associated with smaller follicles and corpora lutea resulting in low peripheral plasma concentrations of estradiol and progesterone [41,46]. This would likely result in subfertility in cattle.

## 4. Genital Microbiome and Breeding Management

The genital tract microbiome present when oocytes develop is thought to also influence normal sperm functions and fertilization capabilities, thereby influencing conception [2]. Like bacteria, sperm are deemed foreign bodies by the genital tract immune system and therefore trigger an inflammatory response upon attaching to TLRs. TLR-2 present at the endometrial glands is important because it removes ‘less fit’ and excess spermatozoa, possibly to prevent polyspermy, as well as prepare the uterus for impending nidation [47]. Postpartum, uterine infections are known to disrupt endometrial epithelial integrity and reduce uterine gland functions by disrupting prostaglandin secretion, thereby compromising folliculogenesis and perturbing fertility [40,48,49]. Many reproductive infections are known to impair fertility. If present in the uterus, *E. coli*, *T. pyogenes* exotoxin, and Bovine Herpesvirus 4 (BoHV-4) are known to disrupt reproductive hormone secretion [43,50,51]. For instance, *E. coli* secretes LPS, which increases prostaglandin E2 (PGE2) production by the endometrial glands instead of PGF2α [43]. PGF2α is luteolytic hormone, while PGE2 is a luteotropic hormone that causes persistence of the corpus luteum in absence of a conception, resulting in pseudopregnancy or luteal cysts and anestrus. In addition, progesterone produced by the persistent corpus luteum dampens immune responses, facilitates pathogen proliferation, and impairs fertility [52,53]. The highly pathogenic bacteria *T. pyogenes* produces an aggressive toxin called pyolysin which damages endometrial epithelial cells, leading to dysregulated hormonal secretion and disrupted fertility [28]. Furthermore, low counts of *Corynebacterium*, *Staphylococcus*, and *Prevotella* two days before insemination are known to improve pregnancy rates in cattle [21].

While the beneficial effects of intravaginal *Lactobacillus* are not yet well known, in the gut, it produces phenolics which protect the oocyte against oxidative stress, thus improving oocyte competency and fertilization [54,55,56]. Infusing *Lactobacillus* as prebiotics during cattle breeding would likely improve fertility when better quality oocytes are ovulated and fertilized [54,57]. In addition, *Lactobacillus* suppress the production of eicosanoids, which are implicated in the inflammatory reaction and negatively affect the oocyte quality when produced at high concentrations. In humans, *Lactobacillus delbrueckii* is abundant in the vagina and is known to increase sperm capacitation through the production of bicarbonate ion (HCO_3_^−^) from water and carbon dioxide [58,59]. To improve fertility in cattle, further studies on the benefits of using such probiotics around the time of breeding are warranted. This may result in reduction in number of services per conception and increased conception to first service and higher pregnancy rates.

## 5. Genital Microbiome and Pregnancy

Technologies such as fluorescence in situ hybridization and 16s rRNA gene sequencing allowed the identification of bacterial species in pregnant cattle [11,60]. The detected bacteria are often associated with uterine diseases and abortion; however, the pregnant cows were clinically healthy. Thus, the gravid uterus is not sterile, contrary to what was previously thought [61]. Interestingly, the abundance and diversity of the microbiomes differ widely from that found in diseased endometria. The microbial load and diversity within the genital tract during pregnancy are relatively low, possibly to reduce the risk of dysbiosis and abortion [1,62]. The common bacterial phyla found in the pregnant uterus of cattle, at placentome and inter-cotyledonary placenta, are *Firmicutes* and *Bacteroidetes* [11]. Another study, using fluorescence in situ hybridization, detected an abundance of *F. necrophorum, T. pyogenes*, and *Porphyromonas levii* in the endometrium and placentomes of cows [60]. It seems that the presence of *Lactobacillus* is beneficial for the success of pregnancy. In humans, the presence of *Lactobacillus* was associated with good placental growth and angiogenesis [63]. In addition, *Lactobacillus* decreases the production of proinflammatory cytokines, which improves the immune tolerance of the conceptus and facilitates the nidation process [64]. Same statements were reported for in vitro fertilization where the dominance of the non-*Lactobacillus* microbiome on the endometrium was associated with decreased implantation rate [63]. In pregnant women, the absence of or decrease in *Lactobacillus* population in the vagina reduces the competition effect against pathogens, leading to an overgrowth of more invasive microbes which will trigger an inflammatory reaction associated with the high release of cytokines, mainly Interleukin (IL)-8, and premature delivery or abortion [65]. Within this context, bovine endometrial epithelial cells were cultured with four different *Lactobacillus* strains elaborated with proinflammatory cytokines IL 1A, IL6 and IL8 [66]. The study confirmed that some *Lactobacillus* strains, such as *Lactobacillus ruminis* and *Lactobacillus amylovorus*, increased the cytokine gene expression concomitantly with the increased microbial load. It is possible that low cytokine production stimulated by *Lactobacillus* has immunomodulatory effects. Without *Lactobacillus*, pathogens proliferate, stimulating excessive proinflammatory responses and thereby perturbing fertility. On the other hand, a high abundance of *S. aureus* and *T. pyogenes* in the bovine genital tract were associated with an increased risk of abortion [67,68]. Therefore, dysbiosis of the vaginal and endometrial microbiomes hinders fertility.

## 6. Genital Microbiome and Postpartum Complications

The postpartum period is a critical window of the production cycle of cattle. Perturbed uterine involution can result in metritis, endometritis, or cystic ovarian disease. To optimize fertility, uterine involution should be uninterrupted and completed around 45 days postpartum. However, bacteria that contaminate the uterus during parturition could slow these two events [69,70,71]. These bacteria are originated from the external environment or the animal’s adjacent organs [11,15,60]. Retained fetal membranes (RFM), metritis, and endometritis are the most frequent postpartum complications in cattle. In cows with normal pregnancy and parturition, all females have a similar microbiome at calving that then becomes divergent by seven days postpartum (DPP), when *Fusobacteria* and *Bacteroidetes* predominate in cows that experienced dystocia or RFM [71]. It is thought that the genital tract microbiome during the postpartum period has a profound effect on subsequent reproductive performances [22,72]. RFM is the persistence of placental tissue attached to the endometrium, representing a favorable environment for the growth of pathogenic bacteria, leading to subfertility [73]. Using culture- and molecular-based methods for microbiology identification, *E. coli* (68%) and *S. aureus* (18%) were the most associated to RFM [74]. Furthermore, RFM is an important risk factor for uterine diseases, mainly metritis and endometritis [73,75,76].

Metritis is a deep inflammation of the endometrium and myometrium, which is associated with purulent and or fetid vaginal discharge, detected before day 21 postpartum [77]. Cows with metritis harbor a less diverse microbiome dominated by *Bacteroides*, *Porphyromonas*, and *Fusobacterium* [5,78]. It is thought that the higher abundance of *Proteobacteria* in the vagina seven days before calving predicts the occurrence of metritis postpartum, because it synergizes *Fusobacteria* [71]. *E. coli* is highly detected in uterine samples in the case of metritis, and the severity of symptoms is more important when the disease is induced by *E. coli* strains harboring the virulence factor kpsMTII [79]. In addition, a symbiotic relationship between *E. coli*, *T. pyogenes*, and *F. necrophorum* facilitates colonization of the endometrium and evasion of the immune system, leading to metritis [80]. *T. pyogenes* produce a powerful cytolytic substance, pyolysin, which alters the endometrial epithelial cell membranes, leading to a tissue damage and disruption of the mucosal integrity [28]. While the endometrial cells do not respond to damaged-associated molecular pattern (DAMP), the combination of pathogens and DAMP triggers an inflammatory reaction through the intracellular secretion of IL-1 by the endometrial cells [28].

Endometritis is a superficial inflammation of the endometrium, which is associated with less severe clinical symptoms compared to metritis and detected after day 21 postpartum, as it is considered as abnormally sustained postpartum inflammation [77,81]. Endometritis is highly debated regarding the origin and pathogenesis of the disease. While it is not confirmed that endometritis is triggered by bacteria, rather than uncontrolled uterine inflammation, several microbes were isolated in animals with clinical and subclinical endometritis [28,29,30,72]. Metagenomic analyses of 16S rRNA gene sequences of the uterine microbiome in endometrial cytobrush collected at different times during the first week postpartum revealed that metritis and clinical endometritis are associated with a lower microbial diversity postpartum, dominated by *Bacteroidetes*, *Fusobacterium*, and *Trueperella,* and lower abundance of *Escherichia, Shigella, Lactobacillus, Prevotella, Schlegelella,* and *Streptococcus* [28,29,30,71]. In addition, abundant *Anaerococcus*, *Corynebacterium*, and *Staphylococcus* increase the risk of subclinical endometritis in postpartum cows [28,29,30]. An interesting synergy between *T. pyogenes, F. necrophorum*, and *Prevotella* species would affect the severity of the clinical symptoms of endometritis [82].

Interestingly, the vaginal and uterine microbiome during the postpartum period affect the reproductive performances in subsequent breeding [22]. For instance, gene sequencing targeting the V1 to V3 hypervariable regions of the 16S rRNA bacterial gene in uterine and vaginal flushes confirmed that low abundance of *Corynebacterium*, *Staphylococcus*, and *Prevotella* two days before insemination improve the pregnancy rate [21]. Therefore, it is important to control the genital microbiome before insemination using probiotics to improve fertility.

## 7. Genital Microbiome and Animal Behavior

The microbiome of different organs confirmed to be effective in modulating the animal behavior toward its conspecifics. The most extensive studies regarding pheromone production are conducted in wild animals [83,84,85]. However, it is possible that microbiomes of livestock, including cows, may show similar diversity patterns. It is thought that microbiomes and animal behavior in wild mammals mirror ruminants [26]. For instance, the microbiome and volatile secretions of the anal scent gland were compared between spotted and stripped hyenas [85]. Interestingly, secretions of the scent gland vary according to the detected microbiome. In addition, both volatile and microbiome profiles of the anal scent gland were different between spotted and stripped hyenas and vary according to the reproductive status of the animal, all of which means that the microbiome and by-products of volatile molecules are highly specific to the species and social groups. The same findings were reported in wild meerkats [86]. 

Likewise, trimethylamine is a powerful volatile substance produced by mice and helps in matting. Interestingly, trimethylamine is highly specific to the species, as it attracts mice but repels rats. Specific commensal bacteria of the intestine metabolize the dietary choline to produce trimethylamine [3]. Likewise, the genital microbiome of different animals, including bovines, was confirmed to be implicated in pheromone production and semiochemical signaling between females and males [87]. This is a solid confirmation that any changes in the female’s genital microbiome affect her pheromone profile and other males’ behavior toward the female. Thus, the genital microbiome of an animal does not only modify the organism’s function and volatile profile but also affects the behavior of the other animals, including predator–prey interaction and feeding behavior [88,89]. Likewise, the females of *Anopheles gambiae*, the African malaria mosquito, target their hosts based on the chemical cues released that they produce and the load and diversity of the skin microbiome. Humans with low skin microbial diversity are the target of choice for mosquitos. In addition, the presence of some bacteria (i.e., *Pseudomonas* spp.) repels the mosquitos [88].

Bovine cervicovaginal mucus (CVM) contains abundant commensal microbes and volatile compounds. During estrus, CVM is highly rich in pheromones that are specific to estrus, among which oleic acid, trimethylamine, acetic acid, and propionic acid are the most important [27]. It seems that pheromones are more concentrated in the CVM than any other body secretions. In an interesting experiment, urine, saliva, feces, and CVM of females in estrus were rubbed on the vulva of anestrus females; these females were exposed to males and Flehmen behavior was monitored [87]. Secretions of other females at pre-estrus and post-estrus were also compared, and water was used as a negative control. Interestingly, the CVM resulted in the highest and longest Flehmen behavior compared to urine, saliva, and feces. No Flehmen reaction was reported in the secretions of females that were not in estrus. Furthermore, the CVM of buffalo during estrus is highly rich in *Firmicutes*, which are fermentative anaerobic bacteria [23]. *Firmicutes,* the phylum which includes the *Lactobacillales* order, is abundant in the genital tract of healthy cows compared to those with uterine disease [5,90]. These bacteria are well known to be implicated in the pheromone production in anal scent glands of hyena [85]. Any modification of the genital microbiome by external factors would affect the volatile profile of CVM. Likewise, intravaginal administration of antibiotics in bitches at estrus reduces the attractiveness of males compared to the untreated females [91]. These findings confirm that pheromones are highly produced in the genital tract and are implicated in the sexual communication between males and females. Thus, it is important to reduce antibiotic use intravaginally, since it affects the microbial diversity of the genital tract and the produced pheromones. 

## 8. Genital Tract Microbiome and Probiotics

Metritis is caused by several bacteria, mainly *E. coli, F. necrophorum*, and *T. pyogenes* [5,77,80]. Due to the high economic loss associated with medication, milk withdrawal, and reduced fertility, antibiotics and antimicrobials are often used to control the disease. Third-generation cephalosporins are the molecule of choice for the treatment of metritis. However, many countries such as the USA have banned their use in food animals, to be only reserved for human medicine [92]. In addition, abusive use of antibiotics increased the risk of the development of resistant strains and the accumulation of antibiotic residues in milk and meat [93,94]. Therefore, developing new alternative protocols for a better management of reproductive diseases is a must. The term “probiotics” represents live microorganisms that confer health benefits on the host when they are administered in adequate amounts [95]. The use of probiotics is promising in treating human urogenital diseases. *Lactobacillus* maybe be part of a bacterial resident population in the uterus constituting a protective microbiome. Thus, uterine microbiota and specific bacterial species may be linked to critical health status such as endometritis, improved conception rates, or abortion. Cattle fertility will benefit from expanded studies of genital tract microbiota interactions. *Lactobacillus* are widely used as probiotics for treating different genital or general diseases because they seem to be tolerated by the host. In addition, *Lactobacillus* spp. can modulate the immunity of the host by producing bioactive molecules such as lactic acid and hydrogen peroxide [9,10]. Finally, it seems that these bacteria are highly tolerated by the host organism and are rarely implicated in genital issues. For example, *Lactobacillus* was used to treat vulvovaginal candidiasis, a fungal infestation of the vagina induced by *Candida albicans* [96]. The patients received capsules of *Lactobacillus plantarum* intravaginally for five weeks, which resulted in better resolution of symptoms than in the patients that received clotrimazole alone as intravaginal cream. Likewise, *Lactobacillus crispatus* were successfully used to manage recurrent urinary tract infections, mainly induced by *E. coli* [97]. The patients were treated with intravaginal capsules containing *Lactobacillus crispatus* for 15 days, which resulted in a lower recurrency rate of the disease compared to the control group. 

Furthermore, oral supplementation of mice with *Lactobaccillus rhamnosus* resulted in better swimming test, lower anxiety, and higher expression of γ-aminobutyric acid (GABA) receptors in the brain [98]. GABA is a powerful inhibitory neurotransmitter in the central nervous system that reduces neuronal excitability throughout the nervous system [99]. The partial removal of the vagus nerve, which communicates between the gastro-intestinal tract and the brain, resulted in an obliteration of the effects induced by *Lactobacillus* supplementation. This is a clear statement that all the information of the digestive system including the gut microbiome is transmitted to the brain and affects the animal’s behavior. 

*Lactobacillus* would likely to play a significant role in controlling the postpartum uterine diseases when infused directly intravaginally as probiotics. Incidences of endometrial inflammation postpartum were reduced when *Lactobacillus sakei* and *Pediococcus acidilactici* were introduced intravaginally in animals before and after calving [100,101]. *Pediococcus* are another valuable probiotic, as they have specific genes encoding for Pediocin, a potent bactericidal peptide [102,103]. Likewise, *Lactobacillus buchneri* were introduced intravaginally in dairy cows between 24 and 30 DPP, and the uterine health status and reproductive performances were measured and compared to a control group [94]. Probiotic treatment resulted in shorter median days to first service, lower number of services per conception, higher first service conception rate, and shorter median days to conception compared to the placebo group treated with isotonic saline solution. In addition, gene expression of pro-inflammatory cytokines and chemokines was lower in the probiotic group. These results confirm that the presence of *Lactobacillus* in the genital tract protects the genital health status and improves fertility of cows.

## 9. Genital Tract Microbiome and Prebiotics

Alternatively, favorable conditions that encourage the proliferation of a healthy microbiome as prebiotics can be ensured [61,104]. Providing a high-energy diet around calving affects the diversity of the uterine microbiome and the occurrence of uterine inflammation. For instance, pregnant multiparous Holstein cows were fed with a diet that covered 80% of the energy requirement, while the other group received a diet that covered 100% of the energy requirement, between 20 days pre-calving and 30 days postpartum [104]. As major results, the energy restriction resulted in an abundance of *Bacteroidetes* and *Fusobacteria* phyla and higher expression of inflammatory cytokines in the endometrium. According to a previous study, to switch from a healthy uterus postpartum to a metritic uterus, there must be an overgrowth of *Bacteroidetes* and *Fusobacteria* and reduced abundance of *Proteobacteria* and *Tenericutes* [5]. 

## 10. Conclusions

Various organs have distinct microbial inhabitants. Apart from the gut, the group that has attracted the most attention in livestock research is the one in the genital tract. Altogether, these examples indicate the involvement of genital tract bacteria in improving fertility. While the consequences of reducing bacterial diversity in the genital tract microbiome remain unexplored, intravaginal inoculation of cattle at risk of infection represents possible probiotic management of postpartum uterine diseases. It maybe that probiotics that alter the endometrial microbiome could provide viable alternatives to existing therapies for uterine disease in cattle.

## Data Availability

Not applicable.
